# Improve the cervical cancer prevention behaviors through mobile-based educational intervention based on I-CHANGE model: study protocol for a randomized controlled trial

**DOI:** 10.1186/s13063-022-06744-5

**Published:** 2022-09-24

**Authors:** Sara Kazemi, Fatemeh Zarei, Alireza Heidarnia, Fatemeh Alhani

**Affiliations:** 1 Department of Health Education and Health Promotion, Faculty of Medical Sciences, Tarbiat Modares University (TMU), P.O. Box 14115-331, Tehran, Iran; 2 Department of Nursing, Faculty of Medical Sciences, Tarbiat Modares University (TMU), Tehran, Iran

**Keywords:** Cervical cancer, Prevention behaviors, Mobile-based, Educational intervention, I-CHANGE model

## Abstract

**Background:**

Applications of mobile technologies (mHealth) have the potential to reduce health inequalities, give patients more control over their health, and improve health care’s cost-effectiveness. The widespread use of mobile phones offers us a new way to prevent cervical cancer. The objective of the study was to design and develop a mobile phone application (app) that aims to conduct a behavioral intervention for women and to evaluate the efficacy of the app-based intervention.

**Methods:**

This study involves 5 phases. In the first phase, understanding women’s perspectives will be identified using a qualitative approach based on the I-Change model. In phase 2, the results from the qualitative approach and requirement prioritization through providing experts’ perspectives will be done. The main outputs of this phase will be resulted in prioritizing the main measurable effective variables of the I-Change model. Phase 3 will be processed for the development and psychometric of an assessment tool regarding selected constructs. In phase 4, the App framework and content development will be performed. In phase 5, a three-armed, parallel-design randomized controlled trial will be conducted on women. Two hundred ten women will be randomly assigned to three groups including two intervention groups and one control group. The intervention groups included the following: (1) a mobile application and (2) a digital book. The data will be evaluated using tools designed and constructed in phase 3 of the study at baseline in 3-month follow-up assessments. The impact of the two interventions on cervical cancer prevention behaviors through mobile-based educational intervention will then be evaluated.

**Discussion:**

A theory-based health education program using a mobile app to improve cervical cancer-preventive behaviors will be implemented for the first time in Iran. With an effective health mobile-based educational design, it is very important to determine whether Iranian women will be motivated to adhere to preventive behavior related to CC.

**Trial registration:**

Iranian Clinical Trial Register IRCT20181205041861N3. Registered V2.0 on 26 October 2021.

## Background

Cervical cancer is one of the “gravest threats to women’s lives,” according to the World Health Organization (WHO) [1]. Cervical cancer is a major public health issue and the fourth most frequent cancer among women in the world, with an estimated 570,000 new cases diagnosed each year [2]. According to WHO estimates, cervical cancer will cause 474,000 women per year by 2030 [3]. Cervical cancer is a disease of inequality. Women in low- and middle-income nations are disproportionately affected by cervical cancer [4]. The vast majority of cervical cancer deaths (90%) occur in low- and middle-income countries, despite these nations receiving only 5% of global cancer resources [5, 6]. Cervical cancer can be prevented in the majority of cases if women get screened. Significant gaps in access to care, on the other hand, have moved the disease burden to resource-poor countries in Africa, Asia, and Latin America [7]. CC is a disease that affects all women who have had at least one sexual encounter. This disease is slow-moving, and early identification with a Pap smear can avert serious complications [8].

### Cervical cancer status in Iran

In Iran, the rate of cervical cancer is reported to be 3.73 per 100,000 women [9]. While cervical cancer is not frequent in Iran, patients usually seek medical help when the disease has progressed to an advanced stage [10, 11], and the mortality rate is high [12]. Most Iranian women are unaware of screening tests and Pap smears test (PST) because of their cultural, economic, and social status [13]. Despite the remarkable success of the Pap test in detecting cervical cancer, the participation rate in developing countries is only 5%, compared to about 90% in high-income countries such as the USA [14]. Many studies in Iran have reported a low participation rate in this test. For example, this rate has been reported at 27.1% in Babazadeh et al.’s [15] study [16], 50% in Farzaneh et al.’s [16] study, and 58.6% in Ghalavandi et al.’s research in Andimeshk city [17].

### Global prevention strategy for CC

The World Health Organization (WHO) established a global strategy to speed up the elimination of cervical cancer in November 2020. By 2030, the goal is to test 70% of women by the age of 35 (and again by the age of 45) and treat 90% of those with precancerous changes [18]. To achieve this lofty goal, scalable screening and cost-effective treatment technologies will be necessary [19]. The impact of cervical cancer prevention initiatives is determined by two key context-specific factors: (1) women’s access to screening and (2) successful treatment acquisition for women who test positive. Due to geographic and infrastructure constraints, access to both screening and treatment is particularly difficult in some locations [20, 21].

### mHealth: a great potential for health education

In the previous several years, the use of mobile technologies has increased [22]. Mobile health (mHealth) has a lot of potential in many aspects of health, such as promotion and prevention, because of this greater access to technology [23]. The use of mobile health to deliver health services has the potential to eliminate inequities, empower individuals to take charge of their health, and enhance the cost-effectiveness of health care [24, 25, 26]. Mobile applications have become increasingly essential in the delivery of educational content in recent years [27]. Because people carry their phones with them wherever they go, educational interventions may be offered at any time to everyone, with further help available on-demand, wherever and whenever it is required. This opportunity allows a wide spectrum of people to benefit from simple and low-cost initiatives [28]. Mobile phones can be used to send motivational messages, monitoring, and behavior change tools [29, 30]. The smartphone application was effective in sustaining the effects of the educational program for prevention of sexually transmitted infection [31]. To educate women and encourage them to participate in cervical cancer screening, and thus strengthening grass root level primary health provision, could be a popular and culturally appropriate intervention, which needs to be tested. Furthermore, it may contribute to the implementation of the National Cervical Cancer Screening and Prevention policy and ultimately help to reduce cervical cancer mortality in Iran. The health promotion package through mobile application will be a simple, feasible, and effective approach. This paper explains our study protocol aiming to determine the effect of educational intervention via mobile app for empowering women in preventive behavior in cervical cancer.

## Methods

### Aim, design, and outcomes

This practical randomized trial aims to evaluate the effect of an interventional education based on I-Change model via mobile phone application on improving preventive behaviors for cervical cancer among Iranian women. An exploratory sequential mixed-methods design will be used in the study.

### Outcome measures

The initial research questions addressed in this study are “Does the educational intervention based on the I-Change model via mobile app affect CC preventive behaviors among Iranian women?”. To answer this main question, the following primary outcomes are expected:

1. Determining and comparing the effects of an educational intervention designed based on the I-Change model via a mobile app knowledge related to CC prevention in women in the target and control groups before, immediately, and 3 months after the interventions.
2. Determining and comparing the effects of an educational intervention designed based on the I-Change model via a mobile app on information factors related to CC prevention in women in the target and control groups before, immediately, and 3 months after the interventions.
3. Determining and comparing the effects of an educational intervention designed based on the I-Change model via a mobile app motivation to CC prevention in women in the target and control groups before, immediately, and 3 months after the interventions.
4. Determining and comparing the effects of an educational intervention designed based on the I-Change model via a mobile app predisposing factors to CC prevention in women in the target and control groups before, immediately, and 3 months after the interventions.
5. Determining and comparing the effects of an educational intervention designed based on the I-Change model via a mobile app behavioral intention to CC prevention in women in the target and control groups before, immediately, and 3 months after the interventions.
6. Determining and comparing the effects of an educational intervention designed based on the I-Change model via a mobile app action to prevent for CC prevention in women in the target and control groups before, immediately, and 3 months after the interventions.

Consequently, we expect the effect of our educational intervention on preventive action regarding CC among women. To assess the expected secondary outcomes, a self-reported assessment addressing preventive actions (using a condom, doing pap-test and genital examination) will be conducted.

### Ethical approval

The study protocol has been ethically approved by the Faculty of Medical Sciences, Tarbiat Modares University of Iran (reference number: IR.MODARES.REC.1400.049). The rights and welfare of the participants will be protected according to the Declaration of Ethical Principles for Medical Research. Data that is collected as part of the study will not be linked to any individual, personal identifiers will not be used in data storage, and confidentiality will be maintained at all levels of data management. Only members of the research team have access to data collected in the project. Plans for communicating important protocol modifications (e.g., changes to eligibility criteria, outcomes, analyses) to relevant parties (e.g., investigators, trial participants, trial registries) will be considered and the corresponding author of this research is responsible for any protocol amendments.

### Participation

The research population will include women aged 18–49 years who register interested in participating in a trial intervention. Participation in the study will be stretched to all eligible women until we reach a sample size of 210 women who provide informed consent to participate.

### Requirement

First, participants will be recruited via an online call through the virtual platforms such as Instagram pages, WhatsApp, and Telegram channel. Candidates will communicate with the trained research assistant (SK; the first author) through the phone number included in the announcement. Then, the research assistant explains the purpose of the study by phone for women who are eligible to enter the study. She will obtain contact information from potential trial participants. The assent will be sent via email or any social media messenger such as Telegram or WhatsApp. Table 1 contains a complete list of the inclusion and exclusion criteria.

**Table 1:** Inclusion and exclusion criteria

Inclusion criteria	Exclusion criteria
Women aged 18–49 years Married (having at least one sexual relationship experience) Being the residence of North Iran during the study Tend to participate with informed consent to share information, and participate Women own a smartphone and the ability to use it Women without mental disorders, drug dependence, and addiction	Absence of more than two sessions in training sessions Having a special illness that prevents participants to take part in training sessions Woman who is not interested in the subject of the curriculum

### Randomization

After informed consent and baseline data collection, participants in the randomized controlled trial will be randomly assigned to the intervention and control arms in a restricted randomized block design. A research identification number will be given to each woman. Then, the identified individuals who volunteered to participate in the study were randomly assigned to the experimental and control groups using a blocking method. Following simple randomization procedures, participants will be assigned to one of the three arms of the trial in a 1:1:1 ratio according to a computer-generated randomization schedule via https://www.sealedenvelope.com/simple-randomiser/v1/lists. The letter A will be considered for the intervention group1, the letter B will be considered for the intervention group2, and the letter C for the control group. The process of random allocation will be continued continuously until the sample size will be reached. Masking of participants and study staff is not possible due to the nature of the intervention (educational) and allocation ratio.

### Study design

This exploratory sequential mixed-methods study will be divided into five phases, which are described below.

Table 2 shows the enrollment, interview, intervention, and assessment schedule. This protocol was developed and reported following the Standard Protocol Items: Recommendations for Interventional Trials (SPIRIT), and the clinical trial will be carried out and reported in accordance with the Consolidated Standards of Reporting Trials (CONSORT).

**Table 2:**
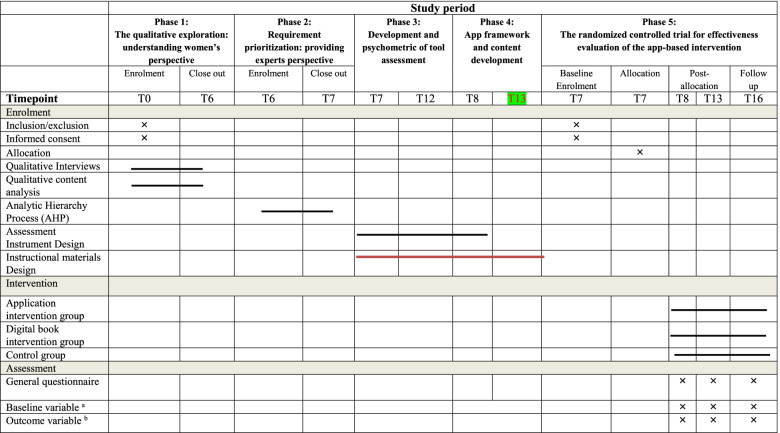
Schedule of enrollment, interviews, intervention, and assessment of the Educational Intervention trial, following the Standard Protocol Items Recommended for Clinical Trials (SPIRIT) guidelines

^a^Age, marital status, number of deliveries, economic status, etc

^b^The main measurable effective variables of the I-Change model

### Procedures

#### Phase 1: the qualitative exploration: understanding women’s perspective

The qualitative research will take 6 months to complete. Understanding the perceptions of women toward CC preventive behavior will be explored using semi-structured interviews with open-ended questions and directed qualitative content analysis methods. The interview will begin with semi-structured questions based on the I-Change model as a theoretical framework [32]. Then the probing questions, following the interviewee’s answers, lead the researcher to explain women’s perceptions and preventive behaviors about cervical cancer prevention. For example, the interview begins with a general question “What have you done so far to avoid cervical cancer?” and continues with guiding questions such as Can you give an example? or “Do you have any experience?”

The interviews will take place face-to-face in a quiet environment that is mutually convenient for both parties. In accordance with the study’s goals and objectives, an interview guide has been created. The first question is a broad, open-ended inquiry about CC preventive behaviors, to which they will be asked to respond in detail. Then, based on the answer, more probing questions will be asked. The goal is to gain a comprehensive understanding of women’s perceptions and behaviors about CC prevention. Each interview will be transcribed in its entirety after it is completed. To ensure that the researcher has accurately interpreted the participants’ statements, the transcripts will be sent to each participant (member check) along with a summary of key topics from each interview. The sample size of the interview process will depend on the information saturation rule which means as the sample expands, no new theme will be induced from the information the participants supplied.

#### Phase 2: requirement prioritization—providing experts’ perspective

Realized findings from phase 1 (QR) will provide all perceptions of participants toward CC preventive behaviors. After all, *the Analytic Hierarchy Process* (AHP) [33] will be run to provide us with a rational framework for a needed decision by quantifying its criteria and alternative options, and for relating emerged themes and constructs to the overall goal. The main outputs of this phase will be resulted in *prioritizing* the main measurable effective variables of the I-Change model [32]. The next step will be processed for the development and psychometrics of an assessment tool regarding selected constructs.

#### Phase 3: development and psychometric of tool assessment

The tool that will be used to collect the data is a questionnaire which will be designed based on achievements from previous phases. Then, the initial tool will be ready for the psychometric process.

#### Phase 4: app framework and content development

Based on our previous studies and references reviewing, preliminary functional modules of the app will be collected. The next step of this study is to search and evaluate currently existing domestic cervical cancer/HPV prevention-related apps on App Store and Android Market. We use the words “HPV/ sexually transmitted disease/Cervical Cancer” respectively to search for related apps. Each eligible app will be downloaded and its features, including names, download numbers, target population, and main function modules, will be listed and analyzed. Personal face-to-face interviews with periodical experts and focused group sessions will hold to discuss the framework of the app from the interactivity, feasibility, acceptability, and user experience aspects. Finally, the functional modules of the app will be determined. The application that is going to be made is based on the Android platform, and since the main language of the Android operating system is Java, it will be made with the Java programming language. The app will be developed by a company that specialized in mobile application development. After the app development is completed, 30 participants will be invited to test it, and necessary modifications will be made according to their advice.

#### Phase 5: the randomized controlled trial for effectiveness evaluation of the app-based intervention

##### Intervention description

**Fig. 1:**
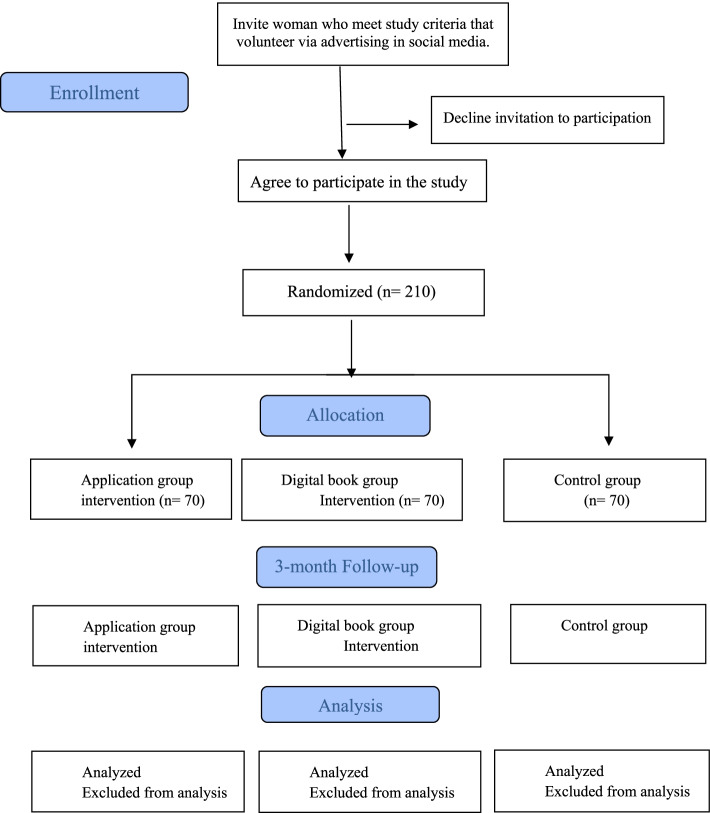
The flow chart of the randomized controlled protocol

This study is a parallel-group randomized controlled trial. Eligible participants are randomized in a 1:1:1 allocation ratio to one of three groups: two experiment groups, in which women receive the application (app) and digital book. The training contents in both experimental groups outlined preventive behaviors of CC regarding I-Change model constructs retrieved from phases one and two. In another word, group A will be called mobile app group and group B will be called the Digi booklet-group and will receive the same training content in different ways (Fig. 1).

Each of the participants in experiment groups A and B will be required to register as a user to download the mobile-App and Digi booklet separately. The educational content will be based on the latest national and international guidelines for the prevention of CC of information (facts).

##### Control group

Group C as the control group will receive no training content during the study. Following the ethical issue, they only will be provided with educational materials at the end of the intervention procedure. They will receive routine care, such as free health information brochures and free counseling services as needed.

### Sample size and power calculations

The sample size was determined by the primary objective (comparison of participation in the experiment and control groups, respectively) and women will be allocated in equal numbers to the three randomization groups. Following the study conducted [34], the sample size we require in the quantitative phase to provide sufficient power was calculated as 61 persons for each group. This sample size was calculated to be adequate at an alpha of 0.05 and a power of .80, to test for a difference between the groups. We considered a potential dropout rate of 15%. According to the formula below, we should start with a recruitment target of 70 participants in each group: ${n}_1={n}_2=\frac{\left({s}_1^2+{s}_2^2\right){\left({z}_{1- {\propto }\!\left/ \ {2}\right.}+{z}_{1-\beta}\right)}^2\ }{{\left(\mu {\mu}_2\right)}^{2.}}$

In line with this approach, a total of 210 women aged 18–49 years will be recruited using the inclusion and exclusion criteria listed in Table 1. Participants will be allocated into three groups (the two intervention groups plus one control group).

### Statistical methods and analysis

#### Phase 1: analysis of qualitative exploration

Phase 1: To perform the process of qualitative content analysis, the audio file of each interview will be listened to attentively several times on the same day and it will be transcribed verbatim. To keep the data from the interviews confidential, a code will be assigned to each transcript. To come up with a general impression of the interviews and become fully immersed in the data, the audio files of the interviews and the transcripts will be reviewed several times, and any possible ambiguities and inconsistencies will be removed by comparing the audio files and the transcripts. The interviews will be audiotaped and a summary of the key issues in each interview will be then sent to each participant to ensure that the researcher will have accurately interpreted that participant’s comments [35]. The process of data analysis will be performed continuously and simultaneously with the data collection process. All words, sentences, and paragraphs that are related in the analysis process will be considered as a single semantic unit. After merging the semantic units, the codes will be extracted; the code together forms the subcategories and then the main categories. Finally, by abstracting the categories, they are placed in I-Change model structures. MAX.QDA software [36] will be used to manage the data.

#### Phase 5: analysis of intervention outcomes

To ensure that the data is correctly entered, a double-entry of data will be performed by two different individuals. All data obtained from women will be entered into SPSS software. A code will be defined to identify missing data. The obtained data will be kept strictly confidential and will be stored with secured and restricted access; also, the signed consent will be kept locked. Follow-up data are received using the same questionnaires as used at baseline. The intervention will be evaluated based only on Pap smear completion 3 months after the enrollment and baseline survey. Data will be summarized with descriptive statistics (mean, median, standard deviation, percentiles for numerical variables, frequencies, and percentages for categorical variables). To determine the normality of data, Smirnov and Kolmogorov will be used. Determining the frequency of demographic information in both groups is analyzed using descriptive statistics templates. To analyze the quantitative variables, the independent *t*-test and paired *t*-test are analyzed. To compare the mean score, the quantitative (intra-group) variables of the two groups of the test and control groups before and after the intervention were used as independent samples. To compare the means, the quantitative (inter-group) variables were compared between the two groups of control and test before and after paired *t*-test intervention is used. In the case of non-normalization, the mean score of the quantitative (intra-group) variables in the two groups of the test and control groups is used before and after the intervention in Mann-Whitney. To compare the meanings, the quantitative variables (inter-group) were used in both control and test groups before and after the Wilcoxon test. To determine the relationship between the underlying and dependent variables in the case of normal, the Pierson correlation test is used and the Spearman correlation test will be used to determine the relationship between the underlying and dependent variables in the case of normal. To analyze the qualitative variables, a chi-square test is used to analyze the qualitative variables. The significance level in this study is considered to be 0.5. The information obtained will be used by the related statistical software, including SPSS version 23.

## Discussion

This study aims to assess the effect of the I-change model on choosing the cervical cancer prevention behaviors in Iranian women. Cervical cancer is a major public health disease in the north of Iran [37]. Even with free screening nationwide and a cervical cancer screening registry, more than 40% of eligible women do not adhere to cancer screening guidelines [37].

Many policy interventions have been performed to improve cervical cancer prevention behaviors [13, 16, 37, 38, 39, 40]; however, they were not sustained for a long time. These experiences indicate that designing appropriate interventions for promoting cervical cancer prevention behaviors and encouragement to perform a Pap smear test are necessary [41, 42, 43]. By providing the conditions, facilities, and equipment to facilitate the testing process, and paying more attention to cultural and social factors in cervical cancer and Pap smear planning, interventions, and policies, barriers to Pap testing can be eliminated [7, 44, 45, 46].

There is the widespread use of mobile technologies around the world [47], but there is currently no randomized controlled trial in Iran that describes the use of these technologies for cervical cancer screening. This study will evaluate the impact of mobile technologies on cervical cancer screening and prevention behavior practices in the north of Iran. If found to be effective in increasing adherence to screening, the mHealth intervention strategy may become an important tool for reducing the cervical cancer burden, and its associated morbidity and mortality. This could also be applied in the future for the health promotion and prevention of other major disease conditions. This requires the development of organized educational programs based on theories of behavior change.

## Limitation

Our study may face several limitations. As it is an open study and participants are aware that they are entering into an intervention entitled in cervical cancer prevention behaviors, bias is likely in the study results. Then, a random allocation of participants can help achieve a balance in sociodemographic characteristics. Another limitation of this study is the need to use self-reporting tools, and the possibility of memory error, lack of clarity, socio-cultural concerns, and individual biases affecting the results are inevitable. Last but not least, our study may have dropouts during the enrolment phase of the CONSORT process because some participants might not have access to a smartphone and the time extension for enrollment to achieve the estimated sample size is probable.

## Conclusion

To the best of our knowledge, this paper is the first published paper that describes the protocol of CC with this design through mobile-based educational intervention based on the I-CHANGE model in Iran. We believe that a virtual educational intervention based on change behavioral theory will lead to improve cervical cancer prevention behaviors and increase the intention to do Pap test among Iranian women. This study estimates that an educational intervention regarding socio-cultural issues will result in a more effective intervention rather than traditional routine training about CC prevention.

### Ethical consideration

Throughout the process, all participants will be assured of their anonymity.

### Trial status

The study is ongoing. Recruitment opened in October 2021 and will continue until all women required for the trial are enrolled, planned to be in December 2022. The duration of the study period will be 1.5 years and will be finished in April 2023.

## Data Availability

Not applicable. The manuscript does not report data. The datasets subsequently generated and/or analyzed during the current study may be made publicly available following the conclusion of ongoing research. Requests for data may be made at any time to the corresponding author.

## Abbreviations

CC: Cervical cancer
mHealth: Mobile health
AHP: Analytic Hierarchy Process
PST: Pap smear test
RCT: Randomized controlled trial
CONSORT: Consolidated Standards of Reporting Trials
WHO: World Health Organization
